# Plasma membrane and brain dysfunction of the old: Do we age from our membranes?

**DOI:** 10.3389/fcell.2022.1031007

**Published:** 2022-10-06

**Authors:** Mauricio G. Martín, Carlos G. Dotti

**Affiliations:** ^1^ Cellular and Molecular Neurobiology Department, Instituto Ferreyra (INIMEC)-Consejo Nacional de Investigaciones Científicas y Técnicas (CONICET), Universidad Nacional de Córdoba (UNC), Córdoba, Argentina; ^2^ Molecular Neuropathology Unit, Physiological and Pathological Processes Program, Centro de Biología Molecular Severo Ochoa, Consejo Superior de Investigaciones Científicas (CSIC), Universidad Autónoma de Madrid (UAM), Madrid, Spain

**Keywords:** aging, membrane, lipids, receptors, signaling

## Abstract

One of the characteristics of aging is a gradual hypo-responsiveness of cells to extrinsic stimuli, mainly evident in the pathways that are under hormone control, both in the brain and in peripheral tissues. Age-related resistance, i.e., reduced response of receptors to their ligands, has been shown to Insulin and also to leptin, thyroid hormones and glucocorticoids. In addition, lower activity has been reported in aging for ß-adrenergic receptors, adenosine A2B receptor, and several other G-protein-coupled receptors. One of the mechanisms proposed to explain the loss of sensitivity to hormones and neurotransmitters with age is the loss of receptors, which has been observed in several tissues. Another mechanism that is finding more and more experimental support is related to the changes that occur with age in the lipid composition of the neuronal plasma membrane, which are responsible for changes in the receptors’ coupling efficiency to ligands, signal attenuation and pathway desensitization. In fact, recent works have shown that altered membrane composition—as occurs during neuronal aging—underlies reduced response to glutamate, to the neurotrophin BDNF, and to insulin, all these leading to cognition decay and epigenetic alterations in the old. In this review we present evidence that altered functions of membrane receptors due to altered plasma membrane properties may be a triggering factor in physiological decline, decreased brain function, and increased vulnerability to neuropathology in aging.

## Introduction

The cellular surface has a critical role in the perception and transmission of external stimuli through transmembrane receptors, although not exclusively. By means of signal transduction mechanisms, these transmembrane receptors determine cytoplasmic and nuclear responses. In the cytoplasm, the activation of membrane receptors amplifies the incoming stimulus by recruiting and modifying the activity of a network of enzymes, resulting in a precise response. In the nucleus, a transcriptional profile is regulated in response to environmental clues, by activation of transcription factors and epigenetic mechanisms that regulate gene expression. Thus, the machinery required for the cell to adapt to the new environment is synthesized.

The function or activity of integral membrane receptors is strictly dependent on lipid-protein or bilayer-protein interactions. These interactions together influence protein conformational transitions that underlie the normal function of membrane receptors. Indeed, as has been clearly described by Andersen and Koeppe (2007), the lipid bilayer works as an allosteric regulator of membrane protein function: i.e., it regulates the receptors’ activity without directly modifying the active site. In addition to regulating receptor activity, the lipid context determines the subcellular localization of membrane receptors and regulates the dimerization ability, lateral diffusion and endocytosis of integral membrane proteins, and their interaction with other adaptor proteins and downstream effectors (Andersen and Koeppe, 2007). Naturally, key aspects of cell pathophysiology will depend on the correct lipid composition of the plasma membrane (Casares et al., 2019).

Aging affects the plasma membrane of all the cells of the body, not only its composition and structure but also the function of its different components. The changes in the lipid bilayer of the plasma membrane are central to all the others, since this is core for the distribution, organization, and function of membrane proteins in all three planes, i.e., within the plane of the bilayer, on the extracellular side, and on the cytoplasmic side. This means that any change in the lipid composition of the cell membranes will impact the function of membrane receptors and the way the cells sense the environment. This is in line with the gate theory of aging proposed two decades ago, based on the observation that age-related quantitative and qualitative alterations in cell surface growth factor receptors, EGFR and PDGFR, account for the diminished proliferative capacity and overall functional deterioration of senescent cells (reviewed by Yeo and Park, 2002). The theory was based on the observation that decreased signaling through EGFR and PDGFR could be explained by the defective endocytosis of ligand-bound receptors, as observed in senescent cultured human diploid fibroblasts (Reenstra et al., 1996), which in turn could be due to reduced functional caveolae (Park et al., 2000; Kawabe et al., 2001).

Numerous studies have shown the existence of significant differences in the relative amounts of the different lipids in tissues of young and old individuals, and this is mainly evident in the brain, because the blood-brain barrier strictly controls the entrance of lipids to the central nervous system. In particular, an increase in saturated fatty acid content and a decrease in polyunsaturated fatty (PUFAs) acid content was found with age starting from 50 years old (Cabré et al., 2017). Arachidonic acid (AA, 20:4 ω-6) and docosahexaenoic acid (DHA, 22:6 ω-3) have been found to be reduced during physiological aging in the cortex and hippocampus of the human brain (McNamara et al., 2008; Cutuli, 2017), although other studies in the human frontal cortex suggested that AA and DHA decay occurs only at later stages in aging (Cabré et al., 2017). Region specific changes in the brain were also described by Favréliere et al. (2000) who found a marked DHA decrease in hippocampus whereas phosphatidylethanolamine (PE), DHA and AA were considerably lower in frontal cortex. Other evident lipid changes described in the brain are a reduction in the content of cholesterol, and changes in the ratios of sphingolipids, gangliosides, and phosphoinositides in hippocampus and other brain regions (Prinetti et al., 2001; Svennerholm et al., 2002; Martin et al., 2008; Haughey et al., 2010; Trovò et al., 2011).

Lipid changes in the old seems to be more pronounced in lipid raft fractions of the plasma membrane. Using lipid rafts isolated from human frontal cortex in nondemented subjects aged from 24 to 85 years, Díaz et al. (2018) showed that these lipid rafts undergo significant alterations of specific lipid classes with aging. The main changes were observed in plasmalogens, AA and DHA, phosphatidylinositol, sphingomyelin, sulfatides and cerebrosides, and cholesterol and sterol esters. Accordingly, several of the same lipid changes in lipid rafts have been reported in specific regions of the aging brain (Colin et al., 2016; Egawa et al., 2016). Furthermore, lipid rafts seems to be particularly sensitive to aging and decreased AA and DHA levels in lipid rafts may represent an early event during normal aging, at least in the brain, contributing to what they called “lipid raft aging” (Díaz et al., 2018).

These results clearly show that lipid remodeling occurs at the plasma membrane with aging. In the brain, some of these changes seem to occur early, at the starting point of the aging process, supporting the hypothesis that reshaping of the plasma membrane may be a very early event in the development of cellular aging, responsible for the occurrence of some of the typical manifestations of aging. However, it should be taken into account that lipid changes are not ubiquitous for every tissue, and different results can be observed depending on the region and the lipid studied.

## Main factors triggering membrane alterations in aging

Considerable evidence indicates that membrane alterations in the old are a normal consequence of metabolic function during the lifespan. The free radical theory of aging (FRTA) (reviewed by Zs.-Nagy, 2014) appears to explain very well how certain lipids, for example PUFAs, become downregulated during aging. Reactive oxygen species (ROS), the byproducts of aerobic life, are particularly harmful to double bonds containing fatty acids (Zs.-Nagy, 2014). During the aging process, free radical accumulation results in membranes with reduced levels of DHA (Horrocks and Yeo, 1999).

Various studies shed light on how these defects could happen. Reactive aldehydes, mainly 4-hydroxynonenal (4-HNE) and 4-hydroxyhexenal (4-HHE) are formed from the breakdown of the products of the peroxidation of ω-6 (mainly linoleic acid and AA) and ω-3 (mainly linolenic acid and DHA) PUFAs, respectively. These 4-hydroxyalkenals seem to be responsible for most of the oxidative damage associated with aging (Riahi et al., 2010). Bacot et al. (2007) demonstrated that phosphatidylethanolamine (PE) is a main target for the reactive aldehyde, 4-hydrohyalkenal, and exposure of different cell models to high levels of 4-hydroxyalkenal affect the structure of biological membranes and its function. Using molecular dynamic modeling, it has been suggested that active PE adducts, the product of PE modification by reactive aldehydes, may be primarily responsible for changes in the lipid membrane properties, such as membrane order parameter, boundary potential and membrane curvature, causing alterations in the function of membrane proteins, like the transport activity of Mitochondrial Uncoupling Protein 1 (Jovanovic et al., 2015). In addition, the formation of PE-alkenal adducts may also affect phospholipase-dependent signaling, as such PE-derived adducts are poor substrates for secreted phospholipase A or phospholipase D (Guichardant et al., 1998).

Direct alterations in protein function induced by 4-hydroxyalkenals posttranslational modification have also been demonstrated. Babych et al. (Babych et al., 2021) found that HNE accelerated pancreatic islet amyloid polypeptide (IAPP) self-assembly into β-sheet fibrils and enhanced the ability of the peptide to permeabilize the plasma membrane and to induce the death of pancreatic β-cells, a pathological hallmark of type II diabetes. Another example of damage induced by 4-hydroxyalkenals on protein function was presented by Choi et al. (2018), who showed that exposure to 4-HNE decreased the function of the cardiac ion channel hERG (human ether-a-go-go-related gene) in primary myocytes.

Decreased levels of DHA and AA in the hippocampus of aged rats (McGahon, 1998) seem to be due not only to lipid peroxidation but have also been attributed to altered fatty acid metabolism (Terracina et al., 1992) and altered levels of fatty acid desaturases (McNamara et al., 2008). Terracina et al. (1992) showed that metabolism of fatty acids is remarkably altered in old brains. They showed that, in 24-month-old rats, there is a significantly decreased incorporation of AA into lipids while the substrate, arachidonoyl-CoA, accumulates by about 50%. It was proposed that the accumulation of arachidonoyl-CoA may have a potent detergent effect, interfering with membrane structure and membrane physiological functions.

Another consequence of oxidative stress in aging is the loss of cholesterol from neuronal membranes. We and others have observed that ROS accumulation induces the expression of the gene *cyp46A1* encoding the enzyme cholesterol 24-hydroxylase, mainly involved in the elimination of cholesterol from the brain (Ohyama et al., 2006; Martin et al., 2008; Sodero et al., 2012). Increased expression of *cyp46A1* in old hippocampal neurons, both *in vivo* and in aging models *in vitro*, results in a consistent 20%–25% reduction of cholesterol in these cells, leading to changes in lipid raft organization (Martin et al., 2008; Sodero et al., 2011).

Increased levels of sphingomyelin (SM) were also identified during aging in the mouse hippocampus and in hippocampal neurons aging *in vitro* (Trovò et al., 2011). In particular, it was shown that the percentage and content of fatty acids longer than 22C esterified to SM increased considerably in cerebral hemispheres, cerebellum and medulla oblongata plus pons in old rats (Giusto et al., 1992). These results point up the long fatty acid tails in SM and ceramide as a factor contributing to increased membrane viscosity or packing density in the aging brain.

Because of the above changes, the membrane viscosity, packing density and thickness undoubtedly change in old cells. For example, increased microviscosity of the lipid bilayers of brain cortical synaptosomal membranes and decreased lateral diffusion of membrane proteins were observed during aging, indicating a rigidification of the membrane structure in old cells (reviewed in Zs.-Nagy, 2014). Early experiments designed to study the effect of these reactive aldehydes also showed that 4-HNE and malonylaldehyde (MDA) decrease mitochondrial membrane fluidity (Chen and Yu, 1994). Consistent with this, recent work from our laboratory (Martín-Segura et al., 2019) demonstrated that hippocampal neurons aging in culture exhibit a greater number and size of highly organized domains. This was revealed through staining with laurdan, a membrane fluorescent dye sensitive to local membrane packing (Golfetto et al., 2013).

## Changes in membrane composition in aging alter hippocampal function

### Membrane lipid changes contribute to brain insulin resistance in old mice

Martín-Segura et al. (2019) showed that both insulin receptor (IR) and insulin-like growth factor 1 receptor (IGF-1R) are constitutively activated in old neurons. Fluorescence resonance energy transfer (FRET) experiments showed that a change in receptor conformation triggered by reduced membrane cholesterol favors ligand-independent autophosphorylation and activation (Martín-Segura et al., 2019). Moreover, a higher number of active IRs was found in the detergent-insoluble membrane domains (or raft fractions) in the hippocampus of old mice (Martín-Segura et al., 2019), suggesting that the reduction in cholesterol enhances receptor-receptor interaction in the plasma membrane. Thus, increased interaction between receptor molecules would induce their autophosphorylation and activation, mimicking the effect of its natural ligand (Lemmon and Schlessinger, 2010; Kavran et al., 2014). However, constant activation of the receptor due to membrane lipid changes will lead to desensitization of the pathways and therefore a lack of response. Consistently, a decline in insulin function during aging was observed as a progressive impairment from insulin-dependent long-term depression (I-LTD), an electrophysiological parameter underlying memory formation. Since it was possible to improve impaired I-LTD by cholesterol addition, and cholesterol depletion in young neurons was sufficient to induce insulin resistance, these experiments strongly indicated changes in the membrane properties of neuronal cells during aging as a main determinant in the decay of synaptic plasticity and cognition that occurs at this late stage of life.

Increased activity of IRs and IGF-1R in the hippocampus of old mice was accompanied by increased activity of their downstream PI3K/Akt pathway (Martín-Segura et al., 2019). Reduced insulin response in a cellular background of high levels of active receptors and PI3K/Akt phosphorylation is considered one of the mechanisms behind insulin desensitization (Zick, 2001). Consistently, Martín-Segura et al. (2019) found that the activity of mTOR (a PI3K downstream target), and the inhibitory phosphorylation of insulin receptor substrate 1 (IRS-1) were higher in the hippocampus of old mice and could be reversed by replenishing cholesterol in the membrane. At this point, it is worth remembering that the inhibitory phosphorylation of IRS proteins by Ser/Thr insulin-stimulated kinases is part of a negative-feedback control mechanism leading to insulin signaling termination. Thus, enhanced PI3K-mTOR activity and inhibitory IRS-1 phosphorylation seem to be part of a mechanism leading to impaired response to insulin in old neurons (Tremblay and Marette, 2001).

**FIGURE 1:**
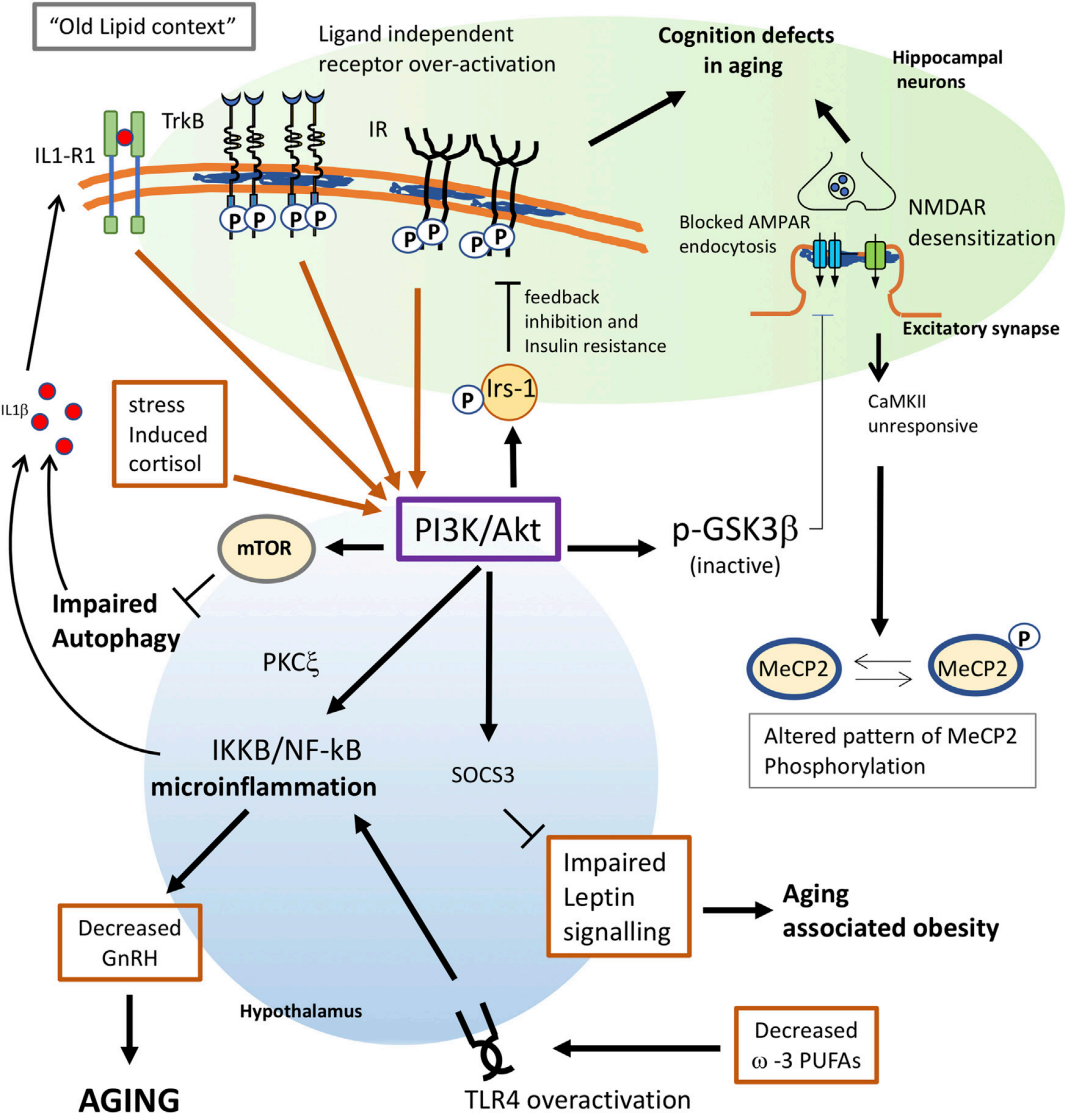
Proposed mechanisms for membrane triggered aging in neurons and hypothalamus. Changes in lipid composition give raise to an “old membrane”. In hippocampal neurons, this old lipid context favors receptor subunit interaction and ligand independent activation of TrkB and Insulin receptors. Constitutive activation of these receptors greatly increases the activity of the PI3K/Akt pathway. Enhanced PI3K will have multiple consequences in old cells: 1) phosphorylation of IRS-1 favors a feedback inhibition of IR signaling, leading to insulin resistance. 2) In neuronal cells, insulin resistance also leads to impaired insulin-triggered memory formation. 3) Akt inhibits GSK3b activity and impairs the AMPA receptor endocytosis required for proper synaptic plasticity. 4) Overactivation of mTOR by PI3K leads to impaired autophagy which, in old individuals, contributes to trigger inflammation and aging. 5) Increased PI3K in the hypothalamus appears to activate NF-kB, leading to microinflammation and decreased secretion of gonadotrophin releasing hormone (GnRH), which is a main determinant for aging. 6) decreased ω-3 PUFAs in old cells or impaired function of G protein-coupled receptor GPR120 would both contribute to trigger proinflammatory signals through overactivation of TLR4. 7) Enhanced PI3K activity in the hypothalamus may impair leptin signaling via Socs3, leading to aging-associated obesity. 8) Stress-induced levels of cortisol would increase AKT signalling. 9) The inflammatory context reached by PI3K overactivation will result in the synthesis of proinflammatory cytokines as IL1β, which further contributes to activate PI3K as part of a positive pro-aging loop. 10) Impaired response of the NMDAR-CaMKII pathway due to lipid alterations will alter the pattern of MeCP2 phosphorylation in old neurons leading to changes in the genome structure.

In summary, these results reveal that one reason for diminished insulin function in the aged brain is increased PI3K/Akt activity and IRS-1 inhibitory phosphorylation, in part due to the constitutive activation of tyrosine kinase receptors - IR and IGF-1R - all of these processes being triggered by cholesterol loss (Figure 1).

### Membrane lipid changes contribute to ligand-independent activation of tropomyosin-related kinase B signaling in the old

Further evidence that membrane receptors can be activated by protein-lipid interactions arises from studies on TrkB receptors in the mouse hippocampus. Tropomyosin-related kinase B (TrkB) receptor signaling, triggered by the interaction of this receptor with its endogenous ligand brain-derived neurotrophic factor (BDNF), is a core participant in the neuronal plasticity process. In earlier studies, we demonstrated a direct link among aging, cholesterol loss, and TrkB activation in hippocampal neurons. We showed that, similar to what was found for IR, the age-related decrease of cholesterol in hippocampal neurons was accompanied by the recruitment of TRKB to membrane-resistant microdomains (rafts), promoting receptor-receptor interaction and ligand independent autophosphorylation, both *in vivo* and *in vitro* (Martin et al., 2008). The pharmacological reduction of up to 25% in membrane cholesterol in primary hippocampal neurons was able to activate TrkB and its downstream effector Akt (Martin et al., 2008; Iannilli et al., 2011; Martin et al., 2011). Similarly, cholesterol replenishment in old acute hippocampal slices and in primary neurons with low cholesterol content led to decreased p-Akt levels (Martin et al., 2011; Martin et al., 2014). Later Trovò et al. (2011) showed that this pathway is chronically activated when the SM/cholesterol ratio increases, further supporting the notion that chronic activity of membrane receptors can occur in the absence of ligand, simply because of changes in its lipid content (Figure 1).

Supporting these results, a cholesterol-recognition amino acid consensus motif (CRAC) was found in 2019 in the transmembrane region of the tyrosine kinase receptor family (RTK) in several species using *in silico* methods (Fantini and Barrantes, 2013). An inverted version of CRAC (CARC), also identified as a cholesterol binding motif, was found in TrkB in vertebrates (Cannarozzo et al., 2021; Fantini and Barrantes, 2013). In 2021, Casarotto *et al.* found that cholesterol switches TrkB dimer orientation between two conformations: one competent and other incompetent for signaling.

The above results demonstrate that membrane lipid remodeling with age is able to modulate membrane receptor activity, what could explain some of the many other defects in hormone and neurotrophin sensitivity that characterize this stage of life. Below we give some examples based on this possibility.

### Altered membrane composition in old neurons impairs glutamate signaling and memory formation

Changes are required in the number and composition of postsynaptic AMPA and NMDA glutamate receptors (AMPARs and NMDARs) to induce and consolidate memory formation (Malenka, 2003; Plant et al., 2006). For AMPARs, these changes occur through membrane-associated processes such as endocytosis, exocytosis, and rapid lateral diffusion between synaptic and extra-synaptic compartments (Choquet and Triller, 2003; Groc et al., 2004; Petrini et al., 2009). Heine et al. (2008) showed that, after fast consecutive synaptic stimulation, the recuperation of a basal state requires fast AMPAR exchange of desensitized receptors with naive functional ones near the postsynaptic density, and this process occurs by lateral diffusion. Experimental data showed that, if surface AMPAR movement is prevented, these interventions directly impact synaptic transmission (Heine et al., 2008). Thus, it is not surprising that membrane AMPAR mobility could be impaired in aged membranes due to lipid alterations. In fact, we showed that this directly occurs in aging hippocampal neurons due to cholesterol loss (Martin et al., 2014). We found that AMPA receptors are efficiently internalized from the plasma membrane of hippocampal neurons maintained in culture for 2 weeks, but that this mechanism fails in neurons maintained in culture for 3 weeks or more, coinciding with the presence of numerous signs of aging. By quantum dot labelling of the AMPA receptor on the neuron surface, we directly demonstrated that aging *in vitro* induced the deficient internalization of AMPARs due to impaired lateral diffusion out of the synapse, and that this in turn is due to the constitutive loss of cholesterol from the plasma membrane (Martin et al., 2014). Consistent with the fact that impaired AMPAR mobility will contribute to cognitive deficits in old individuals, electrophysiological recordings demonstrated that impaired long-term depression in the old may be improved by cholesterol perfusion. Furthermore, cholesterol infusion in the lateral ventricle of old animals improved hippocampal-dependent learning and memory in the water maze test (Martin et al., 2014).

A complementary mechanism that would explain how the loss of cholesterol may contribute to the cognitive deficits in the elderly is the observation that cholesterol depletion triggers the detachment of the PI(4,5)P2-clustering molecule, myristoylated alanine-rich C-kinase substrate (MARCKS), from the neuronal plasma membranes (Martin et al., 2014). MARCKS detachment releases PIP2, which is phosphorylated by PI3K to PIP3. These events result in an increased PIP3/PIP2 ratio in old neurons (Trovò et al., 2013) with several consequences: first, increased PIP3 triggers Akt phosphorylation, leading to the inactivation of GSK3ß, which is required for AMPA receptor internalization (Figure 1). Second, a lower concentration of PIP2 in turn reduces PLCγ activity, with a negative impact on learning and memory (Trovò et al., 2013). Third, it has been shown that PIP3 accumulation as a consequence of increased PI3K activity stabilizes F-actin blocking AMPARs at the dendritic spines (Renner et al., 2009; Martín et al., 2014). These results further indicate that the natural occurrence of cholesterol reduction during aging can contribute to the cognitive deficit phenotype of the elderly.

The work of other laboratories has also demonstrated the importance of appropriate cholesterol levels in the plasma membrane for the glutamate receptors to exert their functions. Thus, it was shown that the distribution and function of NMDA receptors (NMDAR) depends on the lipid environment, as cholesterol depletion prevents NMDA-dependent Ca^2+^ influx in cultured hippocampal pyramidal cells (Frank et al., 2004) and inhibits NMDA-induced long-term potentiation (LTP) in the hippocampus (Frank et al., 2008). In addition (Korinek et al., 2015), showed that cholesterol depletion significantly diminishes NMDAR responses and increases NMDAR desensitization in cultured rat cerebellar granule cells.

In summary, all these evidences indicate that the response of neurons to glutamatergic stimuli will not be the same in neurons of old individuals as in young ones, and that part of the responsibility for these changes is due to the reduced cholesterol levels in the plasma membrane.

## Endocrine/neuroendocrine decline during aging—Could it be due to membrane alterations?

The decline in the responsiveness in endocrine/neuroendocrine systems during aging was reported, and discussed, many years ago by Scarpace (1988) and others. For example, parathyroid hormone stimulation of 1,25-dihydroxyvitamin D production declines with age in the kidney as does calcium absorption in the intestine; decreased responsiveness of ß-adrenergic receptors was observed with age in the myocardium; and memory dysfunction was found in old rodents partly due to decreased muscarinic-cholinergic receptor function.

Although the reduction in receptor activation during aging has been generally attributed to receptor desensitization due to exposure to increased agonist concentrations, this mechanism could not fully account for the reduced responsiveness in any of these systems. For example, parathyroidectomy does not restore 1,25-dihydroxyvitamin D production nor intestinal calcium absorption. Similarly, desensitization due to increased levels of neurotransmitters could not explain completely the defects found for muscarinic receptors in the old. It was found that, for these receptors, desensitization requires phosphoinositol-dependent conformational changes triggered by the agonist. These conformational changes lead to a status of high affinity for the ligand, accompanied by a decreased response to the agonist. However, changes in the binding characteristics of muscarinic receptors were observed in hippocampal membranes from senescent rats for agonists that weakly stimulate phosphoinositol turnover and do not induce conformational changes in the receptor (Scarpace, 1988). One explanation for this could be that the changes in phosphoinositol levels found during aging (as reported in Trovò et al., 2013) may impact muscarinic-cholinergic receptor conformation, leading to memory deficits in old individuals.

**FIGURE 2:**
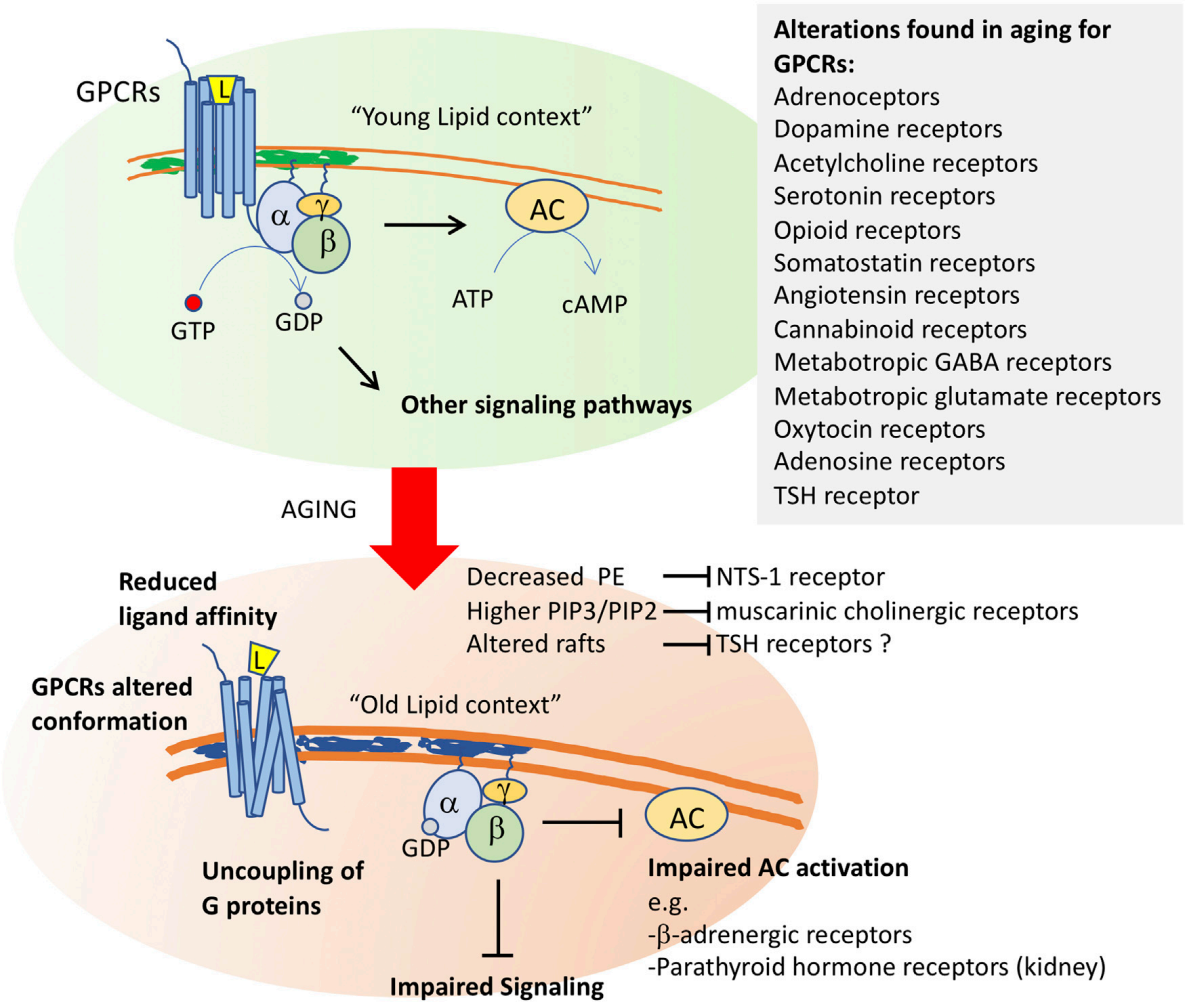
Membrane aging leads to G protein-coupled receptor dysfunction. After ligand (L) binding, the G protein-coupled receptors transduce environmental signals by promoting a GTP-GDP exchange at the α-subunit of the coupled trimeric G protein. Activated G proteins then initiate signaling through several pathways, including the activation of adenylyl cyclase (AC). Changes in membrane properties in old cells impact GCPR function at several levels. It will alter the conformation of these receptors, whose quaternary structure is formed by 7-transmembrane segments. Altered function was shown for neurotensin receptor-1 (NTS-1) by decreased levels of phosphoethanolamine, for muscarinic cholinergic receptors by altered phosphoinositide ratios, and it has been proposed for thyroid stimulating hormone receptor, whose proper activity occurs in raft fractions of the plasma membrane. Altered membrane composition uncouples G proteins from their receptors, impairing downstream signaling. Impaired AC activation in aging membranes was shown for b-adrenergic receptors and for parathyroid hormone receptors in kidney. Alterations in the function of a high number of GPCRs (listed in the inset) have been shown in old cells in several models.

Another factor that could account for the decreased response of these three types of receptors is a decreased ability to activate their coupled adenylate cyclase due to membrane alterations. In support of this possibility, it was found that parathyroid hormone receptors are less able to activate adenylate cyclase in old rat kidneys and ß-adrenergic receptors have a lower ability to activate adenylate cyclase in most tissues with aging (Scarpace, 1988; Liang et al., 1990; Xiao et al., 1998). This was observed for several other G protein-coupled receptors (see below and Figure 2).

### Membrane alterations reduce G protein-coupled receptor signaling with age

G protein-coupled receptors (GPCRs) belong to the largest family of transmembrane receptors, with over 800 members in the human species. Alterations in the expression and activity of GPCRs have been observed during normal aging, and particularly affect the central nervous system (de Oliveira et al., 2019). Decreased expression or activity of GPCRs and G proteins has been associated with alterations in neuronal plasticity and increased sensitivity to neurodegenerative processes, with a consequent decline in cognitive, motor, and even sensory capabilities. Thus, GPCR signaling decline appears to be a critical factor in cognitive aging and in age-related pathologies (Alemany et al., 2007).

Among the causes leading to alterations in GPCR activity or density in aging, the altered structure or function of the plasma membrane lipids or changes in membrane fluidity by lipid peroxidation have been proposed (Alemany et al., 2007). For example, reduction in serotonin 5-HT1 receptor density with aging could be rescued by Gingko biloba treatment probably due to the reversion of the altered lipid environment of neuronal cell membranes, i.e., the increase in viscosity and reduction in fluidity due to lipid peroxidation (Huguet and Piriou, 1994). Similarly, Dijkman and Watts (2015) demonstrated that, in lipid environments rich in phosphatidylethanolamine (PE), the Gαi1 subunit has a ∼4-fold higher affinity for the GCPR neurotensin receptor 1 (NTS1) than in the absence of native lipids. As commented above, PE is one of the main targets of lipid peroxidation, and remarkable decreases of PE levels were found in old membranes. In addition, a decrease of a specific Gi/o-coupled GPCR signaling with aging could also be due to alterations of membrane lipids and other GPCR interactors at the plasma membrane, all composing a functional receptorsome (see de Oliveira et al., 2019).

Aging alterations have been reported in several other GPCRs, including adrenoceptors, dopamine receptors, acetylcholine receptors, serotonin receptors, opioid receptors, somatostatin receptors, angiotensin receptors, cannabinoid receptors, metabotropic GABA receptors, metabotropic glutamate receptors, oxytocin receptors and, as we will discuss below, adenosine receptors (de Oliveira et al., 2019) (Figure 2).

### Adenosine receptor desensitization in the old

The adenosine A2B receptor (A2B) is a G-protein-coupled receptor abundantly expressed in skeletal muscle as well as in brown adipose tissue. Gnad et al. (2020) showed that specific ablation of A2B in these two types of tissue recapitulated important aspects of aging, including reduced muscle strength and mass, as well as reduced brown adipose tissue-dependent energy expenditure. In contrast, stimulation of A2B counteracted aging effects in these tissues. In addition, the markers of cellular senescence, caveolin1, activin A, p16, and p21 were significantly upregulated in the absence of A2B. All these data suggest that alterations in the G-coupled A2B signaling would contribute to triggering the aging phenotype.

As was observed for the majority of G-protein-coupled receptors, adenosine receptors undergo agonist-induced desensitization (Mundell and Kelly, 2011). Agonist-induced desensitization has been attributed to different causes. Following prolonged or repeated exposure to agonist ligands, receptors can desensitize, leading to a loss of responsiveness. Homologous desensitization involves the uncoupling of agonist-occupied receptors from their associated G proteins followed by internalization (Gurevich and Gurevich, 2006; Moore et al., 2007; Hanyaloglu and Zastrow, 2008; Marchese et al., 2008). Since desensitization limits the ability of these receptors to couple to intracellular signaling, the evidence suggests that desensitization of A2B receptors in old cells may account for the alterations observed in aging-related defects. But what is the cause of A2B receptor desensitization in old cells? Is it related to age-associated membrane lipid changes?

Albeit not directly proven, the presented evidence suggests that G protein uncoupling from A2B receptors due to alterations in membrane lipids followed by enhanced endocytosis would be one mechanism behind the changes in A2B receptor signaling in aging. Naturally, this is an aspect that should be investigated directly.

### Thyroid hormone resistance with aging

Age-related resistance to thyroid hormone **(**TH), thyroxine (T4) and triiodothyronine (T3) has been widely reported. Old subjects with normal plasma levels of TH can show symptoms of hypothyroidism and patients with excess of TH can be asymptomatic (Mooradian, 2008). The symptoms accompanying age-related resistance to THs have been attributed to decreased TH transport to tissues, decreased activation of T4 to T3, and decreased nuclear receptor responses. Mechanistically, resistance to TH occurs both at the level of the thyroid gland and on the neurons of the hypothalamic-pituitary axis (Mooradian and Wong, 1994) yet the underlying mechanisms seem to be different in nature. One of the mechanisms proposed to explain central TH resistance is that aging of the pituitary decreases thyroid stimulating hormone (TSH) bioactivity, for example because of altered TSH glycosylation (Jansen et al., 2015; Oliveira et al., 2001). However, and in line with what we have discussed above, decreased activity of the TSH receptor in the thyroid may be due to changes in membrane properties with aging. Supporting this possibility, it was shown that the TSH receptor is constitutively associated with lipid rafts (Latif et al., 2003). Upon activation with increasing concentrations of TSH, the ligand-receptor complexes move out of the rafts to initiate signal transduction (Latif et al., 2003). This leads us to speculate that some of the thyroid hormone resistance with age may also be explained by the changes in membrane lipid composition leading to receptor desensitization or decreased activity of their G coupled proteins, as was proposed for other hormone receptors.

### Decay of growth hormone signaling in aging

Growth hormone (GH) plays important roles in body homeostasis: it promotes lipolysis, and regulates carbohydrate metabolism, cardiovascular system function, aerobic exercise capacity and cognitive function. Circulating levels of GH decline with age in various mammalian species, including humans (Sonntag et al., 1980; Kuwahara et al., 2004; Veldhuis, 2008; Bartke, 2019). In general terms, the age-related decline in GH levels is considered as a determinant factor of certain features of the old, as a consequence of neuroendocrine aging. Consistently, an increase in GH in older adults by a variety of interventions including exercise, administration of GH, or treatment with GH secretagogues, has been shown to increase bone density and muscle mass and decrease adipose tissue. On the other hand, there is an opposite view that, in addition to the effect of promoting reproductive life and improving performance, GH also leads to aging. Thus, observations in animal models have shown that impairments in GH signaling due to mutations affecting anterior pituitary development, reduced GH secretion, or mutations in GH receptors, slow down aging and extend longevity (reviewed by Bartke, 2019). Similar observations were obtained in double mutant mice lacking both GH and functional GH receptors (GHR), which showed extended lifespan compared to controls (Gesing et al., 2016). Coschigano et al. (2003) found that life span was significantly extended for GHR −/− mice but remained unchanged for transgenic mice expressing a GHR antagonist, suggesting that the degree of blockade of GH signaling can lead to dramatically different phenotypes. In terms of cognition, some works have reported that mice bearing impaired GH signaling show improved cognitive function into advanced age compared to wild type controls (Kinney et al., 2001). Similarly, although not in an aging context (Basu et al., 2017), described a negative impact of excess GH and a beneficial effect of the inhibition of GH action on spatial learning and memory in transgenic mice.

Although it is not easy to reconcile the two types (occasionally opposed) of consequences that the decrease in GH has, the fact that the levels of this hormone do decrease with age and that strategies to increase GH levels in adult humans with deficiencies of this hormone have had some success at bone, muscle and adipose tissue levels, it appears reasonable to speculate that, sustained over time, reduced GH signaling with age may contribute to the development of different types of deficits, including, perhaps, cognitive deficits. Until there are experiments that prove this possibility, we will have to settle for keeping this at the level of hypothesis. Nevertheless, what is known about the mechanism behind the decrease in GH signaling with age? Is there evidence to support the possibility that deficiencies in this hormone are due to changes in the plasma membrane?

Evidence indicating an important role for lipid bilayers in the activation of GHR was published more than 20 years ago, when Goldsmith and collaborators (Goldsmith et al., 1997), reported that one of the trafficking pathways taken by GHRs is ligand-mediated accumulation of these receptors in a detergent-insoluble (rafts) fraction of the membrane. Studies developed to understand the mechanism of receptor activation have led to a model in which this receptor exists as a constitutive dimer. Binding of the hormone realigns the GHR subunits by rotation and closer apposition, leading to the juxtaposition of the catalytic domains of its downstream effector, the Janus protein kinase 2 (JAK2), a tyrosine-protein kinase, placed below the cell membrane (Brooks and Waters, 2010). Bocharov et al. (2018) showed that the transmembrane region of GHR can adopt two alternative dimeric structures that correspond to dormant and active receptor states. These structures interchange *via* allosteric rearrangements of transmembrane helices and extracellular juxtamembrane regions that support coordination between protein-protein and protein-lipid interactions. Once activated, GHR signals are transduced *via* the receptor-associated cytoplasmic JAK2. The major intracellular signaling systems activated by JAK2 in response to GH include the signal transducer and activator of transcription (STAT) five and extracellular signal-regulated kinase (ERK)-1 and -2 pathways. In 3T3-F442A cells, it was shown that 35% of the total plasma membrane-associated GHR is localized in caveolin-cholesterol enriched fractions. In these cells, GH treatment resulted in the activation of ERKs associated with the caveolin-cholesterol rich domains, whereas STAT5 activation was only minimally detected in that fraction. Accordingly, treatment with the cholesterol-depleting reagent, methyl-ß-cyclodextrin, significantly decreased GH-induced ERK activation without affecting STAT5 activity (Yang 2004). Later, Rowlinson et al. (2008) found that perturbing the relative orientations of the two transmembrane helices of the GHR with glycine and proline substitutions also changed the ratio of JAK2–STAT5 to ERK signaling. It has been shown that differential activation of these pathways leads to marked phenotypic differences ((Clayton et al., 1999; Lewis et al., 2004).

**FIGURE 3:**
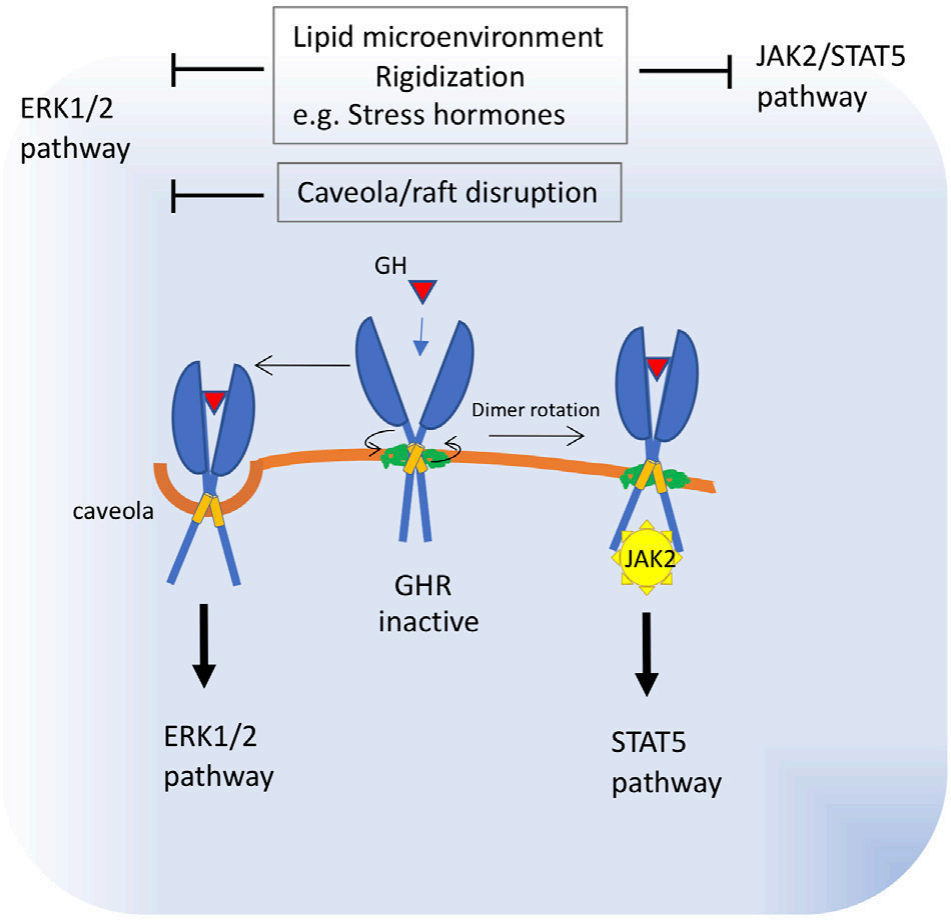
Proposed alterations in growth hormone receptor function in old membranes. The transmembrane region of GHR can adopt two alternative dimeric structures that correspond to dormant and active receptor states. Binding of the hormone realigns the GHR subunits by rotation and closer apposition. These structures interchange via allosteric rearrangements of transmembrane helices and juxtamembrane regions, which are influenced by protein-lipid interactions. Once activated, GHR signals are transduced *via* the cytoplasmic receptor-associated protein tyrosine kinase, Janus kinase 2 (JAK2) and downstream STAT5. A fraction of the GHR population is localized in caveolin-cholesterol enriched membranes and signals preferentially through downstream ERK. These data strongly suggest that GH signaling is influenced by the membrane localization or lipid environment of the GHR receptor. Thus, impaired subunit rotation due to membrane rigidization or increased membrane viscosity will block JAK2 signaling, whereas caveola disruption will impact ERK1/2 signaling. Increased viscosity of plasma membrane was described as a consequence of raised levels of stress hormones.

These data strongly suggest that GH signaling is influenced by the membrane localization or lipid environment of the GHR receptor (Figure 3). For example, GHR localized in the caveolin-cholesterol enriched fractions seems to couple preferentially to ERK activation, whereas those excluded from the caveolin-cholesterol fraction couple to STAT5/JAK2 activation (Yang et al., 2004). In addition, changes in lipid composition that alter the conformation of GHR dimers will have a strong influence on which downstream pathway - ERK or STAT5 - is activated, leading to significantly different responses.

### Leptin desensitization and leptin resistance in aging

Another hormone-dependent signaling pathway that is altered in aging is the leptin pathway. Leptin, a satiety factor synthesized in adipocytes, is considered one of the major signals to control energy homeostasis. Leptin acts in the hypothalamus to control food intake and body weight through a neural circuitry comprised of orexigenic and anorectic signals. Leptin signals through the Ob-Rb receptor and its downstream pathways, which involves the conventional JAK2–STAT3 pathway and the PI3K–PDE3B–cAMP pathway (Sahu, 2003). Leptin resistance, i.e., decreased response to Leptin, contributes to the development of obesity associated with aging. Among the proposed causes of age-related leptin resistance are decreased leptin uptake and downregulation of leptin receptor signaling through the JAK–STAT3 pathway in the hypothalamus. However, it was found that leptin resistance in hypothalamic neurons may occur despite an intact JAK2–STAT3 pathway, indicating the PI3K–PDE3B–cAMP pathway (Sahu, 2003) as a cause of signaling defects in the old. This is because the leptin feedback inhibition mechanism is mediated by the suppressor of cytokine signaling-3 (SOCS3), whose expression can be enhanced by PI3K (Figure 1). Thus, it was shown that aging increases SOCS3 expression in the hypothalamus (Peralta et al., 2002). In the case that increased - yet negative - activity of a tonically active PI3K pathway is present in the hypothalamus, for example due to ligand independent activation of IR or RTK receptors (as was shown for the hippocampus of old mice), PI3K would activate the SOCS3-mediated feedback inhibition mechanisms, blocking leptin signaling in old cells. It will be interesting to know to what extent the mechanisms involved in hippocampal IR and TrkB desensitization in the hippocampus of old mice may also be responsible for age-associated leptin resistance in the hypothalamus.

## Membrane changes may be the cause of a hypothalamic microinflammatory state in the old

The hypothalamus has fundamental roles in the control of several body functions including metabolism and aging. Zhang et al. (2013) have shown that, activating or inhibiting the immune pathway IKKB/NF-kB in the hypothalamus of mice, it was possible to, respectively, accelerate or decelerate the aging process, leading to shortened or increased lifespan. They described a direct link between IKKB/NF-kB activation and gonadotrophin releasing hormone (GnRH) decline, which may result in the end of the reproductive phase and aging.

How the hypothalamic IKKB/NF-kB pathway is activated in early aging in the hypothalamus is not known, but it is not unreasonable to propose that it is due to changes in plasma membrane properties, similarly to what has been described for insulin and TrkB signaling in the hippocampus of old mice. IKKB is a substrate of PKCξ that is activated by the insulin-PI3K pathway, and PKCξ is also involved in the inhibitory phosphorylation of IRS-1. Indeed, it was shown that activation or overexpression of IKKB attenuated insulin signaling (Zick, 2001). Thus, expression of a constitutively active IKKB form in the mediobasal hypothalamus remarkably impaired Akt activation and PIP3 increase in response to an injection of insulin (Zhang et al., 2008). Therefore, we can speculate that IKKB overactivation in the old could be the result of tonically active IR/IGF-1R (as observed in Martín-Segura et al., 2019) and that, although it desensitizes the pathway for the function of insulin on synaptic plasticity, it produces a high activation of the PI3K/Akt branch of the pathway. In addition, the inflammatory context reached by PI3K overactivation would lead to the synthesis of proinflammatory cytokines as IL1β through IKKB/NF-kB (Figure 1), which further contributes to activate PI3K as part of a positive pro-aging loop (Reddy et al., 1997; Sizemore et al., 1999; Chandrasekar et al., 2005; Cahill and Rogers, 2008).

Later, in line with the findings of Zhang et al. (2013), Cai and Khor (2019) proposed the hypothalamic microinflammation theory of aging, speculating that hypothalamic microinflammation emerges during the early stages of aging and metabolic syndrome. One way this could happen is through the activation of the transmembrane receptor toll-like receptor 4 (TLR4) in the hypothalamus. TLRs are expressed on cell surfaces that recognize mainly microbial membrane components and trigger inflammatory responses. Among these, TLR4 is able to activate both MyD88-dependent and MyD88-independent pathways, expressing inflammatory molecules and type I interferons, respectively. TLR4 is also an activator of IKKB/NF-kB (Hayden and Ghosh, 2008).

One of the mechanisms that would trigger TLR4 activation in old cells may involve decreased levels of PUFAs. First, since ω-3 PUFAs exert anti-inflammatory roles (Cintra et al., 2012; Valdearcos et al., 2014), ω-3 PUFA peroxidation in aging would contribute to create a proinflammatory context facilitating TLR4 signaling. Indeed, experimental studies have shown that ω-3 PUFAs inhibit the TLR4-induced signaling (Lee and Plakidas, 2003; Lee and Ye, 2003) probably due to the anti-inflammatory effects of PUFA mediated by the G protein-coupled receptor 120 (GPR120) (Oh et al., 2010). In addition, ω-3 PUFAs disrupt the translocation of TLR4 into lipid rafts, preventing its activation (Wong et al., 2009). Thus, decreased ω-3 PUFAs in old cells or impaired function of GPR120 by G protein-uncoupling (see above) would both contribute to trigger proinflammatory signals through TLR4 (Figure 1). In addition, ω-3 PUFAs mainly EPA and DHA are activators of the peroxisome proliferators-activator receptors (PPARs) (Adkins and Kelley, 2010; Zúñiga et al., 2011), a family of ligand-activated transcription factors which act as NF-κB antagonists (Delerive et al., 1999; Delerive et al., 2000; Delerive et al., 2001; Okayasu et al., 2008). It means that decreased levels of ω-3 PUFAs in aging would also contribute to enhance NF-κB activity through this pathway.

Interestingly, it was found that TLR4 mediates lipid-induced insulin resistance (Shi et al., 2006), providing another link among membrane aging, inflammation, IKKB/NF-kB activation, and insulin resistance.

All together, these data indicate that changes in the plasma membrane lipids may play an essential role in the aging of the hypothalamic-pituitary axis, and from there, in the entire organism.

## Increased stress hormones in aging may alter membrane fluidity and signaling

Further contributing to support the view that changes in the lipid composition of the plasma membrane play a major role in aging are the data showing that microviscosity alterations due to incorporating cortisol into the lipid bilayer may play a fundamental role in regulating signal transduction pathways, including stress-activated cascades (Török et al., 2014). Working in rat erythrocytes, Panin et al. (2010) found that corticosteroids increased membrane microviscosity in lipid–protein domains. They showed that the interaction of hormones with the membrane is accompanied by structural transitions in membrane proteins and in phospholipids, leading to the formation of large lipid-protein and lipid-lipid clusters and distortion of membranes properties.

In agreement with these findings, Dindia et al. (2012) found that stressor-mediated elevation of corticosteroid levels triggers membrane microdomain (rafts) reorganization of liver plasma membrane modulating signaling pathways in rainbow trout (Dindia et al., 2012; Dindia et al., 2013). These authors showed that treating trout hepatocytes with physiological stress-induced levels of cortisol led to phosphorylation of PKA, PKC, and AKT substrates (Figures 1, 3).

Altogether, these results suggest that biophysical changes to plasma membrane properties triggered by stressor-induced glucocorticoid elevation act as a nonspecific stress response and may rapidly modulate acute stress-signaling pathways. This is an important finding in the context of aging, where elevated levels of cortisol in old individuals are associated with higher levels of psychosocial stress, poorer cognitive performance, and atrophy in memory-related structures of the brain such as the hippocampus (Lupien et al., 1998).

## Changes in lipid composition of the plasma membrane lead to epigenetic dysregulation in aging

Changes in membrane properties during aging will not only lead to changes at the proximal level, i.e., in membrane-cytoplasmic homeostasis, but also will have a deep impact in the structure of the genome, since most of the signaling pathways activated by hormones and neurotransmitters are able to regulate the activity of the enzymes involved in the epigenetic regulation of gene expression.

In neurons, for example, proper NMDAR-dependent activity is required for gene transcriptional regulation in response to neuronal stimulation (Tian et al., 2010). After excitatory neuronal stimulation, the activation of ionotropic glutamate NMDA receptors (NMDARs) leads to synaptic Ca^2+^ influx, which in turn activates the kinase CAMKII and downstream transcription factors, such as cyclic AMP response element-binding protein (CREB) (Sheng et al., 1991; Cohen et al., 2015). Activated transcription factors then recruit epigenetic machinery, i. e, histone acetyl transferases and histone demethylases, to modulate the expression of their target genes, including genes involved in synaptic plasticity (Palomer and Carretero, 2016). Also, a direct link has been reported between NMDAR activity and DNA methylation (Martinowich et al., 2003; Lubin et al., 2008). Oliveira et al. (2012) found that DNA methyl transferase Dnmt3a2 was robustly and transiently activated by neuronal activity in NMDA receptor-dependent signaling, showing that DNA methylation is required for memory formation. These results suggest that the structure of chromatin in space and time is essential for context-dependent regulation of gene expression in post-mitotic neurons.

In agreement with the cognitive decay observed in older individuals, dysregulation of epigenetic processes has been widely described in the aging brain (Harman and Martín, 2020). In synapses of old neurons, for example, impaired NMDAR-dependent activation of CREB transcription factor was observed (Foster and Kumar, 2002). In line with these observations, impaired H4K9 acetylation was observed in learning regulated genes (Peleg et al., 2010) and increased levels of the repressive histone marks (e.g., H3K27Me3) were found at BDNF promoters in the old, accompanied by decreased expression of this gene in hippocampal neurons (Palomer and Carretero, 2016; Palomer and Martín-Segura, 2016). Furthermore, it was observed that the reduction in histone acetylation in old neurons makes synaptic plasticity genes vulnerable to *de novo* methylation and repression (Barter and Foster, 2018).

But is there any relationship between alterations in epigenetic regulatory pathways and the changes in the plasma membrane that occur with age? Taking as an example the excitatory mechanisms in hippocampus, current evidence indicates that the epigenetic regulation of genes involved in learning and memory is dependent on the proper activity of glutamate ionotropic NMDAR receptors. Korinek et al. (2015) found that cholesterol depletion diminishes NMDAR responses as the result of a reduction of ion channel open probability. In line with these results, in a previous work from our laboratory, we showed that impaired NMDAR-CaMKII response arises from the constitutive reduction of synaptic cholesterol that occurs with normal aging. Indeed, experimental data showed that increasing the levels of neuronal cholesterol in aged neurons was enough to restore NMDA-induced Bdnf expression and chromatin remodeling at BDNF promoters (Martín-Segura, et al., 2016; Palomer et al., 2016; ). These results provide an example of a direct link among membrane lipid composition, NMDAR activity, and epigenetic regulation.

Another example suggesting that impaired NMDAR-CaMKII signaling in old neurons may deeply affect the neuronal epigenome is through methyl-CpG binding protein 2 (MeCP2) phosphorylation. MeCP2 was initially discovered as a transcriptional repressor, able to recruit histone deacetylases and DNMTs, but it also acts as a transcriptional activator by recruiting the transcription factor CREB to specific genes (Chahrour et al., 2008). In addition, evidence suggests that MeCP2 can bind to both methylated and nonmethylated DNA and mediate nucleosomal compaction (Georgel et al., 2003; Nikitina et al., 2007). MeCP2 deficiency results in global changes in neuronal chromatin structure, including elevated histone acetylation and a doubling of histone H1. MeCP2 mutant brains also show elevated transcription of repetitive elements, contributing to generate genomic instability, which is also observed in old brains. Accordingly, Skene et al. (2010) proposed that MeCP2 may not act as a gene-specific transcriptional repressor in neurons, but may reduce genome-wide transcriptional noise in a DNA methylation-dependent manner.

Early works showed that neuronal depolarization triggers Ca2+-dependent phosphorylation of MeCP2, causing dissociation of MeCP2 from the Bdnf promoter and increased Bdnf transcription (Chen et al., 2003; Martinowich et al., 2003). In turn, Zhou et al. (2006) showed that phosphorylation of MeCP2 occurs at serine 421 by a CaMKII-dependent mechanism which regulates activity-dependent gene transcription. According to its role in chromatin organization, Cohen et al. (2011) found that phosphorylation of MeCP2 S421 appears not to regulate the expression of specific genes but rather functions as a histone-like factor whose phosphorylation may facilitate a genome-wide response of chromatin to neuronal activity during nervous system development. Thus, impaired response of the NMDAR-CaMKII pathway due to lipid alterations will alter the pattern of MeCP2 phosphorylation in old neurons and the profile of gene expression strictly controlled by neuronal activity. Due to its histone-like properties, altered MeCP2 phosphorylation would contribute to modify the genomic structure of the old brain (Figure 1).

Furthermore, since phosphorylation of MeCP2 at Ser421 can be also triggered by dopamine transporter (DAT) or the 5-HT transporter (SERT) in the mesolimbocortical brain regions (Hutchinson et al., 2012), alterations in the activity of these receptors with membrane aging will also contribute to generate genomic defects.

It is reasonable to speculate that lipid changes at the plasma membrane occurring in neuronal cells during aging will also influence the activity of numerous transmembrane receptors and their downstream signaling, leading consequently to epigenetic dysregulation in old cells. This would be the case, for example, of GHR. As we commented above, the transcription factor STAT5b controls the actions of GH on growth and metabolism by regulating many GH-dependent genes, among them *Igf*1. Transcriptional activation of GH target genes is accompanied by the binding of STAT5b to their proximal promoters, promoting enhanced histone acetylation, decreased H3K4 methylation, loss of the transcriptional repressor Bcl6, and recruitment of RNA polymerase II to the promoters (Chia and Rotwein, 2010). This means that altered GHR-STAT5 signaling by changes in membrane localization or the activity of GHR, for example due to recruitment to lipid rafts in old cells, will directly impact chromatin remodeling and gene expression of GH target genes in the old.

Another example of how changes in the properties of neuronal plasma membrane with age may affect epigenetic mechanisms is given by the IR. A direct link between signaling through IR and epigenetic regulation was established by Cierzniak et al. (2021), who showed increased global DNA methylation in adipose tissue biopsies of insulin-resistant patients. Similarly, Małodobra-Mazur et al. (2021) found that numerous genes regulating insulin action (PPARG, SLC2A4, ADIPOQ) were hypermethylated in IR adipocytes which appear to be mediated through DNMT1. Similar increased expression of DNMT1 that correlated with obesity and insulin resistance was demonstrated by other authors (Kim et al., 2015; Lee et al., 2019). Indeed, inhibition of DNA methylation promotes adipogenesis (Kim et al., 2015) and improves insulin resistance in obese mice (Kiskinis et al., 2007). It has been shown that the expression and activity of DNMT1 is regulated by several signal transduction pathways, PI3K/PKB being one of the most prominent ones, which induce higher expression of the enzyme in a programmed manner in different biological settings (Kar et al., 2012). Thus, these data strongly suggest that changes at the plasma membrane level, leading to Insulin resistance and PI3K overactivation in old cells—as is the case for TrkB and constitutive, ligand-independent IR activation - will increase DNA methylation, modifying the epigenetic configuration of old cells.

## Membrane receptor desensitization with age: Improved survival at the expense of performance?

Hormones are largely responsible for the integrated communication of several physiological systems, thus modulating cell growth and organ development and function. Although specific hormonal influences must be considered within the context of the entire endocrine system and in relation with other physiological systems, some of the hormonal systems that change with age seem to contribute to successful aging whereas other pathways lead to increased lifespan, although at times at the cost of functional decline. This kind of survival-at-the-price-of-function dichotomy is most noticeable when we look at what happens to PI3K/Akt levels with age. The PI3K/Akt proteins are common effectors to numerous intracellular pathways, many of which are potentiated during aging, as for example the chronic activation/desensitization of receptor tyrosine kinases like the insulin receptor and TrkB (Martin et al., 2008; Trovò et al., 2013; Martín-Segura et al., 2019). Although the activation of this route will facilitate cell survival (Kim et al., 2002; Miyawaki et al., 2009), it will also lead to a decrease in the strength of the mechanisms required for synaptic plasticity. The latter would be because PI3K activation leads to Akt-mediated inhibition of GSK3b activity (Peineau et al., 2007) and, as a consequence, a reduction in the internalization of AMPA-type receptors, and therefore the development of LTD deficits (Martin et al., 2014). Thus, although membrane lipid remodeling in aging leads to deficits in synaptic plasticity, the increase in activation of the PI3K/Akt pathway due to the same membrane lipid changes diverts signaling towards survival, indicating that, perhaps, loss of functionality is the price to pay to increase the chances of staying alive. It will be interesting to study to what extent it is possible to reduce deficits in synaptic plasticity through strategies to prevent membrane lipid changes without this leading to reduced survival. Experiments of inhibition of the activity of the enzyme Cyp46, which participates in the loss of cholesterol with age (Martin et al., 2008), reduced the cognitive deficits of old mice (Palomer and Martín-Segura, 2016), but it was not analyzed whether these mice presented more neuronal death, or if there was more neuronal damage when the treated mice were subjected to stressful situations. Survival is a very robust mechanism of evolution, under the control of numerous pathways, so it is possible that strategies of reducing lipid changes in the old may prevent some of the functional deficits without greatly disturbing survival.

We propose that the activation/desensitization of membrane receptors during aging is due to changes in the composition and intrinsic properties of the cell membranes, as a consequence of the metabolic function. This has been discussed for GHR, TrkB, insulin/IGF-1, leptin, A2B and in general for several G protein-coupled receptors. It means that, even if ROS accumulation may be the initial trigger for aging, the question about how well the organism faces this ROS accumulation will depend highly on to what extent the plasma membrane properties are affected.

Results from our laboratory have shown that membrane changes in hippocampal neuronal aging lead to constitutive activation and desensitization of IR. These events would clearly contribute to the aging process, a conclusion supported by a vast amount of evidence showing that inhibited insulin and IGF1 signaling extends the life span in animals, from *C. elegans*, to *Drosophila*, to mice (Kenyon, 2005). Indeed, it was shown that Klotho, a peptide that functions as an anti-aging hormone in mammals, works by suppression of ligand-stimulated autophosphorylation of insulin and IGF1 receptors, and decreases downstream signaling (Kurosu et al., 2005). This means that constitutive activation of IR and desensitization of the insulin pathway in aging would align with Klotho negative signaling, that although could prolong lifespan, they might reduce functional capacity.

Changes in lipid composition during aging would also affect GHR activity in two possible ways: enhancing GHR activity as a complementation mechanism to counteract the decreased levels of GH found in old individuals, and thus contribute to promote aging and reduce lifespan or, on the other hand, leading to a constitutive reduction in GHR activity that would contribute to achieve successful aging. Intuitively, it is most likely that aging produces different effects on GH signaling in cells with high regenerative capacity and in those, such as neurons, that lack this capacity. It is not difficult to imagine that a compensatory increase in GH could be beneficial for organs and tissues in which aging leads to progressive cell loss, while its reduction could be beneficial for cells with a high metabolic demand, such as neurons and heart cells. In addition, the consequences of GHR modulation by plasma membrane will be different depending on the pathway favored. For example, aging-triggered lipid changes leading to GHR recruitment to raft fractions, as was shown for TrkB and insulin receptors may favor GHR-ERK activation to the detriment of canonic STAT5/JAK2 signaling. On the other hand, lipid-GHR interactions that change the dimer conformation leading to STAT5 activation would have completely different consequences. Therefore, it seems logical that the results of GH activity studies will largely depend not only on the cell type analyzed but also on the age of the cells under study and the lipid composition of the plasma membrane. In any case, it appears obvious that the changes in the plasma membrane that occur during aging would significantly affect the activity of GH and therefore its functions.

In 2005, Hulbert (Hulbert, 2005) proposed the membrane pacemaker theory of aging. This theory emphasizes that the fatty acid composition of membranes is a critical factor in lipid peroxidation and consequently in the rate of aging and determination of lifespan. Indeed, it has been suggested that the specific acyl composition of membranes works as a timer to determinate longevity in different species (Hulbert and Else, 2000). Long-lived animals also have a low degree of fatty acid unsaturation in cell membranes due to decreases in PUFAs and higher levels of less unsaturated fatty acids (Pamplona et al., 2002; Hulbert et al., 2007; Naudí et al., 2013; Cortie et al., 2015), changes that make them more resistant to lipid peroxidation *in vivo* (Pamplona and Barja, 2007).

As a consequence of this theory, it has been proposed that lifespan extension by calorie restriction could partly be explained by changes in membrane fatty acid composition leading to membranes with decreased PUFAs and relatively higher levels of fatty acids containing lower number of unsaturations, which are thus more resistant to peroxidation (Merry, 2002). Thus, the positive effect of calorie restriction would decrease the levels of PUFAs to minimize membrane damage, but increase the levels of other less unsaturated fatty acids, ensuring that adequate membrane fluidity is maintained for proper membrane receptor responses. Indeed, several years ago, it was described that calorie restriction is able to preserve mitochondrial and microsomal membrane fluidity with age in the liver (Yu et al., 1992), in cardiac ventricular muscle (Lee et al., 1999) and in synaptosomes prepared from brain cortex (Wong et al., 2009).

Also, in agreement with the idea that calorie restriction partly reduces aging by restoration of membrane properties, it was found that calorie restriction had no effect on the number of alpha 1-adrenergic receptors or on receptor binding affinity but prevented the age-related decrease in alpha 1-adrenergic agonist-stimulated increase in GTP binding capacity in rat parotid gland (Chen et al., 1997).

Finally, is worth remarking that some studies reported clear gender differences in brain lipid changes with aging, with lipid alterations apparently being more pronounced in women than in men (Díaz et al., 2018) and in female than in male mice (Couttas et al., 2018). These findings would help to explain sexual differences in cognitive decline during aging and in the differential propension to develop (Casarotto et al., 2021), neurodegenerative diseases (Alzheimer’s Association, 2014).

## Concluding remarks

Starting from the basis that the lipids of the plasma membrane play a critical role in intracellular signaling, both from their own signaling capacity and because they control receptor and channel intracellular signaling strength, we favor the notion that the gradual changes in the lipid composition of the neuronal plasma membrane are a primary cause of the cognitive deficits of the old, upstream of numerous other biochemical alterations that accompany aging, including changes in gene expression. In agreement with Hulbert’s membrane pacemaker theory of aging, much of organism aging could be attributed to the cells’ constitutive membrane lipid changes. In addition, the fact that certain metabolic pathways (e.g., insulin) activate mechanisms of synaptic plasticity in the young but in the old are mainly involved in the activation of survival pathways makes us wonder whether the loss of function caused by lipid changes in the membrane is nothing more than a mechanism to guarantee survival. We therefore hope that this review will serve to motivate colleagues to explore to what extent it is possible to modify the changes in the lipid composition of the plasma membrane that occur throughout life so as to reduce the impact on the functional deficits without leading to increased susceptibility to disease, or directly to cell death.
